# Deciphering Immune Responses to Immunization via Transcriptional Analysis: A Narrative Review of the Current Evidence towards Personalized Vaccination Strategies

**DOI:** 10.3390/ijms25137095

**Published:** 2024-06-28

**Authors:** Ioanna Papadatou, Maria Geropeppa, Christina Piperi, Vana Spoulou, Christos Adamopoulos, Athanasios G. Papavassiliou

**Affiliations:** 1 Immunobiology and Vaccinology Research Laboratory, Medical School, National and Kapodistrian University of Athens, 11527 Athens, Greece; iopapadatou@med.uoa.gr (I.P.); mgeropeppa@med.uoa.gr (M.G.); vspoulou@med.uoa.gr (V.S.); 2 First Department of Pediatrics, “Aghia Sophia” Children’s Hospital, Medical School, National and Kapodistrian University of Athens, 11527 Athens, Greece; 3 Department of Biological Chemistry, Medical School, National and Kapodistrian University of Athens, 11527 Athens, Greece; cpiperi@med.uoa.gr (C.P.); papavas@med.uoa.gr (A.G.P.); 4 Department of Oncological Sciences, Icahn School of Medicine at Mount Sinai, New York, NY 10029, USA

**Keywords:** transcriptomics, systems biology, vaccine-induced immune responses, biomarkers, high-risk populations

## Abstract

The development of vaccines has drastically reduced the mortality and morbidity of several diseases. Despite the great success of vaccines, the immunological processes involved in protective immunity are not fully understood and several issues remain to be elucidated. Recently, the advent of high-throughput technologies has enabled a more in-depth investigation of the immune system as a whole and the characterization of the interactions of numerous components of immunity. In the field of vaccinology, these tools allow for the exploration of the molecular mechanisms by which vaccines can induce protective immune responses. In this review, we aim to describe current data on transcriptional responses to vaccination, focusing on similarities and differences of vaccine-induced transcriptional responses among vaccines mostly in healthy adults, but also in high-risk populations, such as the elderly and children. Moreover, the identification of potential predictive biomarkers of vaccine immunogenicity, the effect of age on transcriptional response and future perspectives for the utilization of transcriptomics in the field of vaccinology will be discussed.

## 1. Introduction

Vaccines represent one of the most successful measures in public health medicine, with profound effects on societal and economic prosperity. Among the most common vaccines, several of them have been proven to be exceptionally efficient in preventing infections, disease, and death. Moreover, the induction of protective immunological memory has been well documented for several vaccines [1]. However, there are still important issues to be addressed, as the immune components required for protection from infection have not been defined and the underlying mechanisms that determine the induction and longevity of vaccine-induced humoral response and immunological memory remain to be elucidated [2].

The intricate interplay between innate and adaptive immunity underscores the necessity for a comprehensive approach to obtain a holistic understanding of human immune responses to vaccines. Conventional immunological methods such as enzyme-linked immunosorbent assay (ELISA), enzyme-linked immunospot (ELISpot) and Flow Cytometry have provided significant insight into vaccine-induced immune responses; nonetheless, these methods are insufficient to analyze the full complexity of vaccine-induced immunity as a whole, as they can only analyze the limited components of the immune response that circulate in peripheral blood shortly after vaccination. To address this issue, the utilization of the so called “-omics” methods for the characterization of vaccine responses is becoming more common [3]. More specifically, transcriptional changes shortly after immunization have emerged as a novel immunological method that can characterize immune response at the gene level shortly after immunization, detecting upregulated and downregulated genes [4]. Additionally, gene modules have been formed to delineate biological functions. The integration of systems biology approaches with clinical and immunologic data offer a promising avenue to unravel vaccine-related mechanisms, identify predictive transcriptional signatures of vaccine immunogenicity and reactogenicity and design tailored vaccination strategies [5].

The magnitude and longevity of vaccine immunity vary between individuals, a phenomenon that remains poorly understood. Genetic factors have been shown to affect vaccine efficacy, with studies conducted on twins demonstrating heritability of vaccine responses ranging from 39–89% [6]. Additionally, genome-wide association studies have revealed the impact of single-nucleotide polymorphisms on immunological response to vaccination, while the role of HLA alleles on antibody responses to vaccines such as influenza, measles and hepatitis B is also under investigation [4]. Furthermore, previous exposure to pathogens could affect vaccine immunogenicity, especially for vaccines stimulating recall responses, such as influenza vaccines, as pre-existing immunity has been linked to decreased response to immunization [7,8]. Utilizing omics technologies as a personalized vaccination tool could enhance our understanding of the complexity of how the human immune system as a whole responds to immunization.

This review endeavors to consolidate current insights into transcriptional analyses of vaccine-induced immune responses, focusing on healthy adults, children, and the elderly and including currently licensed vaccines. Our aim is to explore the potential of transcriptomics in evaluating immune responses to immunization, identifying novel predictive biomarkers, and optimizing personalized vaccination strategies, by summarizing the existing literature on vaccine-induced transcriptional responses (Table 1).

## 2. Vaccine-Induced Transcriptional Responses in Healthy Adults: Commonalities and Differences

In recent decades, the field of vaccinology has grappled with the intriguing question of whether immunization elicits a uniform transcriptional response or if each vaccine engenders distinct gene signatures. Addressing this query, a comprehensive study by Hagan et al. integrated transcriptional data across 13 diverse vaccines, revealing a common transcriptional program shared by viral and bacterial vaccines [52]. Specifically, it was demonstrated that most vaccines induce gene signatures associated with innate immunity and plasmablasts on days 1 and 7 post-immunization, respectively. This study revealed four temporal expression patterns: Cluster 1 represented innate immune modules regarding antiviral response, interferon (IFN)-inducible genes, monocyte- and complement-related modules, which were upregulated on days 1 and 3 and were more prominent for live vector and adjuvant vaccines. Cluster 2 included natural killer (NK) cell modules, which were significantly downregulated on day 1 post-immunization. Clusters 3 and 4 contained plasmablasts, B cell and cell cycle gene signatures, whose activity peaked on day 7, corresponding to plasmablast expansion and being more robustly expressed for bacterial vaccines. Despite this shared transcriptional program, notable deviations and considerable heterogeneity in the kinetics of vaccine-induced immune responses were observed, as early innate and antiviral responses on day 1 post-immunization were absent for the varicella-zoster vaccine and induced later for the yellow fever vaccine. Furthermore, the cell cycle signature, typically expressed on day 7 post-immunization for the majority of the vaccines, was not detected for varicella-zoster, smallpox, and polysaccharide meningococcal vaccines. Apart from the common shared transcriptional response to immunization, vaccine-specific gene signatures have been described for several vaccines, which are further presented in the following paragraphs.

### 2.1. Vaccines against Viral Infections

The first studies utilizing a systems vaccinology approach focused on immune response to the yellow fever vaccine (YF-17D), a highly effective live attenuated vaccine with a rate of protection close to 90% [9]. Gaucher et al. reported an early upregulation of B cell and T cell modules on day 1 post-immunization, followed by a relatively late expression of IFN, complement and antiviral pathways, that started being detectable on day 3 post-immunization and peaked on day 7. Interestingly, a coordinated upregulation of transcription factor genes *STAT1 (Signal Transducer and Activator of Transcription 1), IRF7 (Interferon Regulatory Factor 7*) (day 3) and *ETS2 (ETS proto-oncogene 2, transcription factor*) (day 7), which are master-switch genes mediating the induction of innate immune response pathways, such as type I IFNs, the inflammasome and the complement, was detected [10]. 

Following the initial work on the YF-17D, the field of transcriptomics expanded in order to explore the immune response to other vaccines, with a substantial part of the studies focusing on influenza vaccine-induced responses. Profiling of gene expression in response to influenza trivalent inactivated vaccine (TIV) has revealed an upregulation of IFN-related genes and dendritic cell activation on days 1 and 3 post-immunization and an enrichment of modules related to antibody-secreting cells (ASCs) and cell cycle on day 7 [11,12,13]. Nakaya et al. compared transcriptional and humoral response of healthy adults who were immunized with TIV or live attenuated influenza vaccine (LAIV) [14]. LAIV, but not TIV, induced the upregulation of IFN-related genes, overlapping with YF-17D response [10,14]. Moreover, LAIV vaccinees displayed a significant enrichment of genes expressed in T cells and monocytes, while TIV vaccinees displayed a molecular signature of antibody-secreting cells (ASCs) and unfolded protein response.

Research on Hepatitis B Virus (HBV) vaccine-induced responses has been skewed towards elucidating differences in gene expression in responders versus non-responders. Investigation of the transcriptional response to a three-dose HBV schedule identified a total of nine coding genes that were significantly up-regulated in non-responders at all measured time-points and could potentially represent the gene signature of HBV non-responsiveness [23]. These differentially expressed genes (DEGs) encoded proteins involved in host defense, innate immune response, cell metabolism and regulation of adaptive immunity [53,54,55]. Interestingly, according to a multi-OMICS study, baseline differential expression of 40 genes could also discriminate responders and non-responders; these genes were involved in cytotoxic T cell immune response, T cell lineage selection and maturation and cytokine expression regulation of T-regulatory cells [24,56,57]. 

Studies on the Ebola vaccine-induced transcriptional response reported a peak gene expression on day 1 post-immunization, mostly involving IFN-related genes [27,28]. DEGs displayed two distinct kinetics profiles, as fast- and slow-kinetics genes were identified, with the former peaking at day 1 and returning to baseline by day 3, while the latter returned to baseline by day 14–28. Slow-kinetics genes included mostly interferon stimulated genes (ISGs), several of which are involved in inhibiting viral replication and entrance and cell cycle [58,59]. On the other hand, fast-kinetics ISGs mediated host defensive mechanisms [60]. Furthermore, the magnitude of transcriptional responses and gene kinetics was dose-dependent, as low-dose vaccinees displayed not only up to six times fewer DEGs, but also slower kinetics, with several ISGs expression peaking at day 3 instead of day 1 [27,28]. Another recent study reported a prolonged activation of 22 genes until day 14 post-Ebola vaccination, involved in antiviral response, monocyte chemotaxis and activation and T cell development, implying a persistent activation of innate immunity [29].

Zostavax induced the upregulation of IFN-induced antiviral genes on days 1 and 3 after immunization [30]. On day 7 after vaccination, genes associated with antibody production, such as immunoglobulin genes were upregulated, overlapping with meningococcal and seasonal influenza vaccines. Surprisingly, BTMs related to innate immunity were weakly induced by Zostavax, compared to other live-vectored vaccines.

Popper et al. reported a robust transcriptional response to dengue immunization with Dengvaxia in adults, with DEGs peaking on days 8 and 9 [31]. Gene transcripts were categorized into three clusters. Cluster 1 included ISG transcripts that peaked on days 8 and 9; cluster 2 genes, involved in DNA replication and cell cycle, showed the greatest up-regulation on day 14, while cluster 3 comprised genes related to reticulocytes and neutrophils that were down-regulated on day 14. The vaccine-induced transcriptional response was less robust than the response to natural infection, but with significant overlap, suggesting that vaccination may mimic natural infection at a subclinical level. Moreover, a recently licensed vaccine against dengue (Qdenga) was also reported to induce an early transcriptional signature of antiviral innate immunity, including genes and modules involved in IFN-I response and inflammatory response pathways [32].

The rapid development and emergency use of mRNA vaccines against SARS-CoV-2 has been a landmark in the field of vaccinology, with systems biology approaches being utilized to define successful innate and adaptive immune responses to SARS-CoV-2 immunization. On days 1–3 after dual SARS-CoV-2 mRNA immunization, genes related to innate immunity, such as IFN signaling, dendritic cell activation and inflammatory modules were detected [33,34,35]. Of note, Arunachalam et al. reported that responses on day 7 after both BNT162b2 doses had little relevance to other vaccines, and no induction of B cell and plasma cell modules was detected in BNT162b2 [33]. Ryan et al. reported a distinct gene expression among individuals primed with mRNA vaccines or adenovector viral vector vaccine (ChAdOx1-S,) as the latter displayed the enrichment of plasma-cell- and immunoglobulin-related modules on day 1 after the first vaccine dose and the downregulation of genes expressed in platelets and monocytes [36]. The observed plasma cell signature in ChAdOx1-S-vaccinated individuals after the first dose most likely reflected pre-existing immunity to a component of theChAdOx1-S vaccine or vector. Discrepancies in transcriptional response were eliminated on day 1 after the second primary dose and the booster mRNA dose, as all participants displayed enriched IFN-related signatures. Heterologous vaccination with ChAdOx1-BNT162b2 elicited a more robust innate signature than homologous BNT162b2 vaccination, including genes related to IFN response, CXC chemokines, complement and cytokine signaling [37]. Previous COVID-19 impacted the transcriptional responses to SARS-CoV-2 vaccination, as infected individuals displayed a more robust and prolonged activation of DEGs from day 1 to day 7 after immunization, mostly including genes participating in the IFN, TNF and IL-17 signaling pathways [35,38]. 

### 2.2. Vaccines against Bacterial and Parasitic Infections

Data on transcriptional response induced by vaccines against bacterial and parasitic infections are limited. Obermoser et al. profiled the transcriptional response induced by polysaccharide pneumococcal immunization (PPV) and TIV [43]. On day 1 after PPV, a predominance of inflammation modules was documented. Both vaccines elicited a plasmablast response on day 7, with this response being more robust for PPV and including *CD38* and *TNFRSF17* genes, previously described as parts of predictive signatures for YF-17D and seasonal influenza vaccine immunogenicity [9,14].

Li et al. compared plain polysaccharide (MPSV4) and conjugate (MCV4) meningococcal vaccines to YF-17D, TIV and LAIV, reporting three distinct transcriptional patterns early after vaccination [44]. More specifically, on day 3 the anti-polysaccharide response (overlapped by MCV4 and MPSV4) consisted of modules associated with antigen presenting cells, alongside with pro-inflammatory and complement cytokines; protein recall responses (overlapped by TIV and MSV4) reflected monocyte features on day 3, such as TLR signaling, while the primary viral response (YF-17D) induced genes associated with innate immunity and IFN responses. However, discrepancies became less apparent by day 7, when cell-cycle-related modules were shared among meningococcal and influenza vaccines. Another study comparing transcriptional response to MPSV4 and MCV4 reported a similar response on day 7 post-immunization to TIV and YF-17D, with the upregulation of plasma cell gene modules [9,43,45]. Of note, MPSV4 vaccinees displayed a downregulation of gene modules related to switched memory B cells, supporting the hypothesis of polysaccharide vaccine-induced hyporesponsiveness [45,61]. 

A study comparing transcriptional responses to the anamnestic acellular pertussis (aP) vaccine in adults who were primed with whole-cell (wP) or aP vaccine in infancy revealed differences in the transcriptional response of the two groups, as aP-primed individuals displayed elevated expression of pro-inflammatory genes, with *ICAM1* and *NFKBIA* contributing the most to this difference [47]. Interestingly, this difference was attributed to a specific subset of aP-primed individuals who had higher expression of these genes, most likely due to a potential asymptomatic pertussis infection.

Kazmin et al. compared transcriptional responses to malaria vaccination of either three consecutive immunizations with RTS,S/AS01 (RRR), or two immunizations of RTS,S/AS01 after a primary vaccine dose of adenovirus 35 (ARR) vector expressing circumsporozoite protein [48]. Transcriptional responses on days 1 and 6 after each vaccination included inflammation signaling and cell cycle genes, respectively, corresponding to early innate and late plasmablast responses. In ARR-vaccinated individuals, the genes more strongly induced were involved in innate immunity and IFN-I antiviral response. Both ARR and RRR signatures overlapped with YF-17D, implying that RTS,S/AS01 drives a robust IFN-I antiviral response. Interestingly, B cell- and plasma cell-related modules were downregulated following prime vaccination with ARR but not with RRR. Moreover, NK-cell-related modules were downregulated on day 1 after each vaccination. 

## 3. Delineating Predictive Signatures of Vaccine Immunogenicity

**Figure 1:**
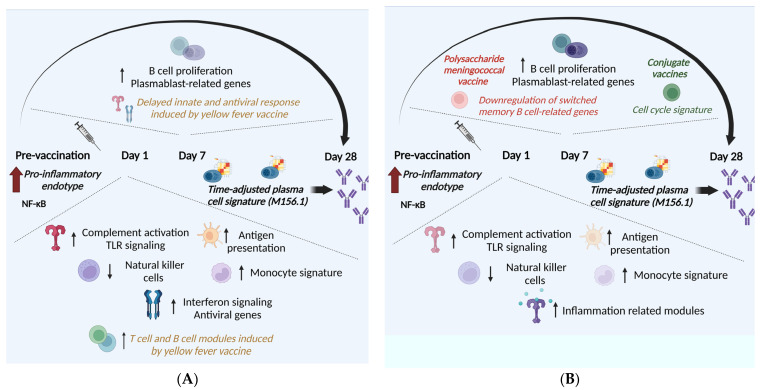
**Transcriptional response to immunization**. A common transcriptional program is shared among vaccines against viral (**A**) and bacterial (**B**) infections. On day 1 following immunization, innate immune responses are induced in most vaccines (most predominant in live viral vector vaccines). Expansion of plasmablast and B cell-related genes are induced on day 7 post-immunization, with this expansion being more robust in vaccines against bacterial infections. However, there are several exceptions in these findings. The yellow fever vaccine displayed a distinct transcriptional profile; on the first day after immunization, T cell and B cell modules were upregulated, while antiviral and innate modules were upregulated later, on day 7 following immunization. Regarding bacterial vaccines, conjugate vaccines were found to stimulate cell cycle signature on day 7, which was not observed for polysaccharide vaccines. On the contrary, the polysaccharide meningococcal vaccine downregulated gene modules involved in switched memory B cell proliferation. Apart from vaccine-specific transcriptional signatures, expression of innate immune modules prior to immunization and a time-adjusted, plasma cell gene signature post-immunization were found to be correlated with subsequent more robust antibody responses to vaccination.

One of the greatest challenges in vaccinology is the identification of biological signatures associated with protective immune responses to immunization. It has been proven for several vaccines that pre-vaccination immune status may have an impact on the subsequent response to vaccination, while vaccine-specific early transcriptional signatures correlating with antibody titers post-immunization have been identified. However, the question whether vaccine-specific or universal predictive biomarkers can be identified either prior to or shortly after immunization remains to be fully addressed. Hagan et al. defined a time-adjusted plasma cell gene signature that could consistently predict antibody responses to 13 vaccines 28 days after vaccination [52]. More specifically, the authors adjusted for time of peak gene expression of this signature; for most vaccines included in the study, this was on day 7 post-immunization, although for some vaccines, such as the YF-17D and the smallpox vaccines, expression of the plasma cell gene signature was expressed much later, on days 10–14 and 21, respectively. Adding to the study, Fourati et al. identified three main endotypes of pre-vaccination transcriptional profiles in a cohort of healthy adults, aged 18–55 years old, namely the “high” pro-inflammatory endotype, including NF-κB, IFN pathways, inflammasome- and monocyte-related genes, the “low” endotype, with decreased expression of innate and IFN-related modules and heightened expression of natural killer cells, B cells and T cells, and the “middle” endotype [62]. Participants expressing a high pro-inflammatory endotype prior to vaccination displayed significantly higher humoral response, suggesting that individuals with a “primed” innate immune system could be at an advantage for rapid response to immunization (Figure 1).

### 3.1. Predictive Biomarkers of Immunogenicity for Vaccines against Viral Infections

Gene signatures predictive of antibody response to vaccination have been identified by several groups. Querec et al. identified a two-gene predictive signature of adaptive responses to YF-17D vaccination with up to 90% accuracy, comprising *EIF2AK4* (involved in integrated stress response) and *TNFRSF17* (involved in plasma cell differentiation) [9]. 

Regarding influenza immunization, both pre- and post-vaccination transcriptional signatures have been associated with subsequent humoral response. A multicohort analysis reported that baseline expression of nine genes and three gene modules in young individuals could predict the magnitude of the antibody response after influenza immunization, including genes involved in B cell signaling (such as *TNFRS17*), dendritic cell and macrophage differentiation and response to inflammation [17,63,64,65,66,67]. Early expression of IFN-related genes and antiviral response modules on days 1 and 7 post-immunization were correlated with antibody response, both in adult and pediatric populations [11,13,14,18,43,68]; of note, these genes were downregulated in children but not in adults on day 3, implying potential age-related differences in the kinetics of immune response.

Accordingly, both pre- and post-immunization predictive biomarkers have been described for the HBV vaccine. Baseline expression of IFN-, granulocyte and the heme-induced response genes has been correlated with attenuated antibody response to HBV vaccination, while the expression of genes related to B cell signaling was associated with stronger responses [24,25,26,39]. These alterations in the heme homeostasis could underpin a hallmark of the hyperimmune inflammation that is observed in the elderly, the so-called “inflammaging” [69]. Moreover, upregulation of IFN-related, pro-inflammatory cytokine, inflammasome and immune cell subset marker genes during primary immunization was associated with subsequent antibody titers [26]. These surprising findings suggest that IFN-related genes may play a dual-faceted role in vaccine-induced immunity, as baseline ISGs expression was inversely correlated with subsequent humoral response, while post-immunization ISGs expression was positively correlated with antibody response.

With regards to the Ebola vaccine, gene signatures of innate immunity on days 1 and 7 and signatures of B cell activation and signaling on day 14 post-vaccination were correlated with a serum humoral response on day 28 [27,28,29]. Li et al. identified post-vaccination predictors of Zostavax vaccine immunogenicity on day 28 post-immunization, including gene expression of plasma cells, immunoglobulins and B cells on day 7, and further emphasized the role of SREBF1 (sterol regulatory element binding transcription factor) and its targets, as their expression was correlated with T follicular response on day 3 and with antibody response on day 7 [30]. The expression of ISGs and cell-cycle related genes on days 8–9 and 14 post-immunization, respectively, were predictive of Dengvaxia immunogenicity [31]. ISG-related gene expression on day 2 after vaccination with Qdenga was also correlated with higher neutralizing antibody titers [32]. Ong et al. reported that early T cell response after MMR immunization may result in attenuated antibody titers [40]. In a cohort of 98 healthcare professionals, non-responders displayed positive enrichment of 10 modules, including 5 modules related to T cell activation and signaling. This early T cell response may prevent or inhibit viral replication and limit antigen presentation and differentiation of memory B cells to plasmablasts, resulting in decreased antibody titers.

Concerning SARS-CoV-2 immunization, Arunachalam et al. identified an early gene signature of monocyte-related modules on day 1 after completion of the primary series that correlated with neutralizing antibody responses [33]. Moreover, antibody responses to a third mRNA vaccine dose were positively correlated with B cell, plasma cell and antigen presentation modules and negatively correlated with elevated expression of neutrophil and inflammation-related modules shortly after the administration of the third vaccine dose [36].

### 3.2. Predictive Biomarkers of Immunogenicity for Vaccines against Bacterial and Parasitic Infections

Obermoser et al. reported a positive correlation of plasma cell, B cell and cell cycle gene signatures on day 7 following pneumococcal immunization with antibody responses, while gene modules related to inflammation displayed an inverse correlation with a subsequent antibody response [43]. Of note, all the correlations were relatively consistent among different pneumococcal serotypes. 

With regards to meningococcal vaccination, early gene signatures of plasma cells, immunoglobulin and memory B cell modules were associated with antibody titers to MCV4, but T cell activation modules were negatively correlated with antibody responses to MPSV4 [44]. Interestingly, the fold change of a single gene transcript, *glucosamine (N-acetyl)-6-sulfatase* (*GNS*) on day 7 post-immunization was significantly correlated with meningococcal serotype C antibody response 28 days after vaccination [45]. *GNS* is a lysosomal enzyme and is found in all cells, while it has been shown to be differentially expressed after YF-17D and TIV [9,14], implying a potential role for this gene in controlling viral and bacterial infection. 

Regarding malaria vaccination, the expression of plasmablast-related modules on day 1 after each vaccine dose, and the expression of antiviral and IFN-I-related modules on day 6 after each vaccination could predict vaccine immunogenicity, while NK-cell-related modules were inversely correlated with antibody titers and protection against infection [48]. Baseline transcriptional signatures, including monocyte pathways, inflammatory response, dendritic cells and cell cycle regulation were correlated with subsequent infection risk [49]. Studies regarding protection from human challenge model infection (HCMI) 14 days after the completion of immunization reported an early up-regulation of the IFN signaling pathway, inflammatory response, apoptosis and the protein kinase cascade on day 1 after the last vaccine dose that remained elevated 14 days after completion of the vaccination schedule in the protected participants, when HCMI occurred [50,51]. These biomarkers could be used for the prediction of malaria vaccine efficacy. 

It is important to note that gene expression perturbation may not necessarily correlate with vaccine efficacy. As demonstrated by seasonal influenza vaccines, efficacy is influenced by multiple factors, including epidemiological data, such as the match between circulating strains and those included in the vaccines each season—factors that omics technologies cannot account for. Additionally, most studies reviewed here focus on vaccine immunogenicity data rather than vaccine efficacy. Future research should be designed to evaluate both vaccine efficacy and to identify predictive biomarkers for both immunogenicity and efficacy.

## 4. The Impact of Age on Transcriptional Response to Immunization

Immune response to vaccination is subject to both vaccine- and host-specific factors. It has been well established that age may have a substantial impact on humoral response following vaccination, as attenuated antibody responses have been reported in elderly individuals compared to younger individuals following immunizations [70]. In this context, it could be postulated that age-related differences are also depicted in the transcriptional response to immunization, a notion supported by several studies.

In reference to influenza vaccination, Avey et al. reported a distinct age-related response to TIV shortly after immunization, as in young adults, an age-associated gene signature was induced 7 days after TIV [15]. Interestingly, this gene signature included *MZB1* and *TNFRSF17* genes, involved in B cell immunity, implying that older adults may fail to mount similar B cell responses after vaccination to young adults. In addition to that, Nakaya et al. reported that older individuals displayed diminished expression of IFN and other innate immune modules compared to younger participants [12]. Furman et al. identified positive and negative predictors of antibody response to the influenza vaccine that were age-related and also reported a reduction in apoptosis-related gene expression in older individuals [16]. The Human Immunology Project Consortium (HIPC) and the Center for Human Immunology were able to identify a baseline gene signature predictive of a response to influenza immunization [17]. Of note, this inflammatory pre-vaccination gene signature was correlated with robust antibody responses in younger vaccinees, but attenuated humoral responses in elderly vaccinees, suggesting that beneficial immune states for vaccination in young individuals may have a detrimental effect in older individuals. Furthermore, Yang et al. described distinct gene modules in an cohort of elderly vaccinees with QIV and identified as a negative biomarker of QIV immunogenicity the gene *MCEMP1* (*Mast-cell expressed membrane protein-1*), which is involved in inflammation pathways [19]. Age has been linked to transcriptional responses in children as well, as older children (9–18 years) displayed increased expression of inflammatory and inflammasome gene modules (up-regulation of IL-17- and NF-κB-related genes in older children) compared to younger children (3–8 years) following immunization with the inactivated influenza vaccine [20]. Interestingly, the authors reported that children who had been previously vaccinated displayed decreased innate gene modules, implying that previous vaccination may result in attenuated innate immune activation, due to decreased antigen uptake and procession.

The response to HBV vaccination may also be influenced by age, as recent studies have underlined. Distinct age-dependent patterns of gene expression were observed after the completion of a two-dose schedule in naïve adults, as baseline upregulation of pro-inflammatory pathways prevailed in elderly participants, including modules of cell motility, the inhibition of integrin signaling, a type II interferon signaling module with expression of TNF and IFNγ, complement genes, and T cell- and NK-cell-mediated cytolysis markers [25]. In addition, Weinberger et al. identified baseline expression of 29 gene transcripts that were associated with age and clustered in a network related to all types of interferons and pro-inflammatory cytokines type I interferons [26]. Age-related gene signatures were distinct during primary and booster immunization; inflammasome markers, IFN-inducible genes and genes encoding pro-inflammatory cytokines dominated the 16-gene signature that separated young and old adults after primary immunization, but after booster vaccination, the 11-gene signature that distinguished young and older adults encompassed genes coding pattern recognition receptors, immune cell subset markers and IFN-inducible genes. 

Li et al. investigated the human response to shingles vaccination with the Zostavax vaccine in a cohort of young adults and elderly individuals [30]. In accordance with findings for other vaccines, baseline expression of gene modules related to inflammation and NK cells was higher in elderly individuals [9].

Age-related differences in transcriptional signatures were also identified following SARS-CoV-2 mRNA immunization, since after the second BNT162b2 dose, younger individuals tended to present greater alterations in monocyte and inflammatory modules, while older participants displayed increased expression of T cell and B cell modules, in accordance with findings for other vaccines [25,33].

These data suggest that there are underlying factors affecting the response of elderly individuals to immunization that warrant further investigation, as a better immunological understanding towards a tailored vaccination strategy is needed for the continuously growing population of elderly individuals.

## 5. Immunizations of High-Risk Populations—What Is Known So Far

While evidence on the immunization of healthy adults is beginning to accrue, there are limited data on transcriptional responses to the immunization of high-risk populations. As far as the pediatric population is concerned, research has mainly focused on influenza vaccination so far. Transcriptomic analysis revealed that both LAIV and TIV vaccines induce the expression of IFN-signaling genes but with differential kinetics; for TIV, over-expression of IFN-inducible genes was observed early on day 1 post-immunization, while this was seen later for LAIV on day 7 [18,21]. A further study by Nakaya et al. comparing MF59-adjuvanted TIV (ATIV) to TIV in children, reported that ATIV induced a more robust and homogeneous transcriptional responses compared to TIV, comprising BTMs related to monocytes, toll-like receptor, antiviral and inflammatory signaling, towards adult-like patterns [68]. In a cohort of HIV-infected, aviremic children, baseline expression of mitochondrial and oxidative stress gene pathways was higher in subsequent low responders to influenza vaccination compared to high responders [22]. Moreover, low responders displayed a prolonged increased IFN and inflammation-related gene expression three weeks post-immunization, compared to high responders. 

Transcriptomics have also been utilized as a predictive tool for vaccine reactogenicity in children. O’Connor et al. described an early neutrophil recruitment gene signature that could detect infants who developed fever shortly after conjugate meningococcal vaccination (4CMenB), underpinning the potential role of neutrophil recruitment in the reactogenicity and immune response to 4CMenB [46]. Moreover, baseline transcriptional data could serve as a predictive biomarker of febrile responses, as a baseline three-gene signature comprising *APBA3*, *AASS* and *FKBP4* could discriminate between febrile and afebrile infants following 4CMenB vaccination. 

A recent study explored the transcriptional response to rotavirus (RV) vaccine and compared these data with children with wild-type infection and healthy controls [42]. Vaccination displayed similar molecular responses with rotavirus infection, as there was an overlap of DEGs involved in gastrointestinal and inflammatory disease and organ injury. In spite of that, no adverse events were reported in the cohort of vaccinated infants. 

Another study utilized transcriptional profiling to investigate the sex-related heterologous effects of measles (MV) and diphtheria–tetanus–pertussis (DTP) immunization in infants, reporting an immunosuppressive effect of DTP on innate pro-inflammatory and T cell responses in females, with down-regulation of genes related to IFN-I signaling and pattern recognition of pathogens [41]. 

Children vaccinated with three doses of RTS,S/AS01 against malaria displayed elevated expression of antiviral and interferon-related modules and decreased expression of monocyte- and antigen-presentation-related modules one month after the completion of the vaccination schedule compared to baseline [49]. Furthermore, an RTS,S/AS01-specific gene signature included four mitochondria and cell-cycle-related BTMs that were associated with protection against infection and three monocyte-related BTMs that were correlated with increased malaria infection risk.

Evidence of transcriptional response to immunization remains very scarce for other high-risk populations. Of note, Kotliarov et al. described the baseline predictive immune signatures of subsequent antibody responses to influenza and YF-17D vaccines in healthy adults, with these signatures correlating with disease activity in lupus patients with plasmablast-associated flares [71]. These signatures included genes involved in the activation of plasmacytoid dendritic cells, switched B cells and T lymphocytes. However, there is a paucity of data regarding the vaccine-induced transcriptional response of populations at risk, such as immunocompromised individuals (individuals with malignancy or immunodeficiencies, transplant recipients, preterm infants, etc.).

## 6. Future Perspectives

Deciphering immune determinants of vaccine-induced protective immune responses could further facilitate rational vaccine design. Transcriptomics can provide essential insight into already known and unknown immune mechanisms, as shown by several studies. Querec et al. identified the *general control nonderepressible 2 kinase* (*GCN2)*, also known as *eukaryotic initiation factor 2α-kinase 4* (*EIF2AK4*), whose expression on day 7 post-immunization could predict the CD8 T cell response to YF-17D [9]. Although the role of *GCN2* in protein synthesis and the regulation of integrated stress response has long been established [72], the identification of *GCN2* as a predictive biomarker for vaccine-induced CD8 T cell response suggested a possible role of *GCN2* in the adaptive response to YF-17D. Moreover, Nakaya et al. described the expression of *calmodulin-dependent protein kinase IV* (*CaMKIV*) gene on day 3 post-TIV, which was inversely correlated with vaccine immunogenicity, while *CaMKIV*-deficient mice displayed increased antibody responses to TIV immunization [14]. These findings suggest an unappreciated role of *CaMKIV* on the regulation of humoral response, as CaMKIV was known until then to be involved in T cell development and responses to inflammation [73,74]. Interestingly, findings from transcriptional studies have identified the potential role of intestinal microbiota in the regulation of immune responses to immunization, as an unexpected correlation between TLR5 gene expression on day 3 after TIV with subsequent antibody titers 28 days post-immunization was reported [14]. TLR5 is a toll-like receptor specific to bacterial flagellin [75]. Future studies may further unravel previously unknown functions of genes in the immune response and guide the development of novel vaccines.

Transcriptional profiling of vaccine-induced responses can also provide essential insight into adjuvants and adjuvanted vaccines biology. Recent studies have identified that oil-in-water emulsions AS02 and MF59 induce a robust up-regulation of immunoproteasome-related genes, implying an increased antigen presentation at the site of injection [50,68]. De Mot et al. reported that alum-based adjuvanted hepatitis B vaccines induced a shared innate gene signature, with differences at the individual level [76]. The application of systems biology approaches in the future could facilitate the evaluation of candidate adjuvants, combining transcriptional and cellular data for the development of optimum vaccine-adjuvant combinations [77]. 

Transcriptomics could serve as a predictive biomarker for monitoring gene signatures in vaccinated individuals and thus for the detection of responders and non-responders to immunization. During the COVID-19 pandemic, the rapid and global deployment of PCR-based assays aided in the diagnosis and treatment of SARS-CoV-2 infection. Based on that experience, simple PCR assays could be developed that can be used for monitoring of vaccine-specific or universal gene signatures in vaccinated individuals. 

In addition, the prediction and detection of subsequent adverse events could be facilitated via the so called “adversomics”. As mentioned above, an early neutrophil recruitment gene signature shortly after conjugate meningococcal vaccination could successfully predict infants that would subsequently become febrile [46]. Twelve gene transcripts on day 9 following dengue vaccination were associated with the subsequent development or rash; these genes were mostly related to IFN pathways [30]. Furthermore, a five-gene signature was identified in one study which was associated with the adverse effect of arthritis following immunization against Ebola [28], including T cell-related genes *CD4*, *CD7* and *GATA3*, the IFN-signaling gene *FCGR1A* and the myeloid-related gene *IL12A*, underpinning the potential role of transcriptomics as a tool for identifying and monitoring vaccinated individuals at risk for developing adverse events. 

The majority of systems vaccinology and more specifically, transcriptomic studies, have focused on healthy adults from high-income countries. However, the target of most vaccines currently used are the extremes of age, with low- and middle-income countries suffering from the main burden of morbidity and mortality from infectious diseases. Moreover, there is a lack of evidence in vulnerable populations, such as immunocompromised individuals, pregnant women, preterm infants and individuals with chronic diseases. Future studies applying systems approaches should be directed to populations that are targets of vaccination schedules, in order to accelerate our understanding of mechanisms underlying vaccine responses in these vulnerable populations.

Although systems biology studies are a promising tool for the profiling of vaccine-induced responses and the characterization of cellular and molecular signatures of immunogenicity and protective immunity, there are several challenges that need to be considered. The majority of transcriptomic studies has been conducted with peripheral blood samples, due to the difficulty of obtaining tissue samples; nonetheless, the characterization of tissue-based responses could provide significant information on the vaccine-induced response at the tissue level and especially at the germinal centers, which are a major orchestrator of the recall response to antigen re-exposure [78]. One must not forget the biological diversity within the human population, as vaccination history, age, chronic comorbidities and environmental factors have an impact on the functions of the immune system [79,80]. A major caveat of high-throughput technologies is the vast amount of data generated with each experiment, resulting in difficulties regarding the extraction of meaningful knowledge from these data in order to improve our biological understanding of immune responses to immunization. From the identification of differentially expressed genes, which is not highly informative and could contain false positives, we have moved on to investigating changes in the activity of biological pathways of modules [81,82,83,84]. However, there is an urgent need for bridging the gap between data and understanding, requiring a combination of biological knowledge, thorough data analysis and most importantly, the collaboration of systems immunologists and bioinformaticians.

## 7. Conclusions

The immune system is incredibly multifaceted. With the progress of technology, we are able to investigate immune responses to the gene level, and transcriptomics will eventually become an essential step in characterizing immune responses to immunizations. More specifically, the combination of traditional immunological methods with the novel omics technologies will help us to decipher the molecular and cellular pathways that dictate immune responses to vaccination, and further understand the differences among vaccine types and adjuvants. Furthermore, identifying novel predictive biomarkers of vaccine immunogenicity shortly after immunization—such as the time-adjusted plasma cell gene signature reported by Hagan et al.—or even before immunization—such as the inflammatory endotype reported by Fourati et al.—as well as age-related predictive gene signatures, including IFN-related and B cell-signaling gene modules, aids in detecting high and low responders to immunization. This knowledge may ultimately pave the way for the design of more effective, tailored vaccination schedules, especially for the protection of high-risk populations.

## Figures and Tables

**Table 1:** Characteristics of the studies utilizing transcriptomics for the description of vaccine-induced immune responses.

Reference	Vaccine (Doses)	Population	Vaccination Schedule (Days)	Bleeding Points (Days)
**Studies on vaccines against viral infections**				
Querec et al., 2009, Nat. Immunol [9]	YF-17D (1)	15 adults (18–45 y.o.)	0	0, 3, 7
Gaucher et al., 2008, J. Exp. Med [10]	YF-17D (1)	15 adults	0	0, 3, 7, 10, 14, 28, 60
Tsang et al., 2014, Cell [11]	TIV (1)	63 adults (>18 y.o.)	0	0, 1, 7, 70
Nakaya et al., Immunity, 2015 [12]	TIV (1)	430 adults (>18 y.o.)	0	0, 1, 3, 7
Bucasas et al., 2011, J. Infect. Dis. [13]	TIV (1)	119 adults (18–40 y.o.)	0	0, 1, 3, 14
Nakaya et al., 2011, Nat. Immunol. [14]	TIV or LAIV (1)	56 adults (18–50 y.o.)	0	0, 3, 7
Avey et al., 2020, J. Immunol. [15]	TIV (1)	317 adults (21–30 y.o., >65 y.o.)	0	0, 2–4, 7, 28
Furman et al., 2013, Mol. Syst. Biol. [16]	TIV (1)	89 adults (20–89 y.o.)	0	0, 28
HIPC-CHI Signatures Project Team, 2017, Sci. Immunol. [17]	TIV (1)	516 adults (<35 y.o., >60 y.o.)	0	0
Cao et al., 2014, J. Infect. Dis. [18]	LAIV or TIV (1)	40 infants, children (6 m.o.–14 y.o.)	0	0, 1, 7, 30
Yang et al., 2020, Front. Immunol. [19]	QIV (1)	16 adults (>60 y.o.)	0	0, 3, 28, 180
Alcorn et al., 2020, Hum. Vaccines Immunother. [20]	TIV (1)	16 children (3–17 y.o.)	0	0, 3, 7
Zhu et al., 2010, Vaccine [21]	LAIV or TIV (1)	85 infants, children (1–3 y.o.)	0	0, 7–10
de Armas et al., 2021, Front. Immunol. [22]	TIV (2)	40 children, adults (4–24 y.o.)	0, 21–28	0, 21–28
Qiu et al., 2018, Hum. Vaccines Immunother. [23]	HBV (3)	14 adults (18–50 y.o.)	0, 28, 56	0 3, 7, 28, 35, 56
Shannon et al., 2020, Front. Immunol. [24]	HBV (3)	15 adults (44–73 y.o.)	0, 28, 180	0, 1, 3, 7, 14
Fourati et al., 2016, Nat. Commun. [25]	HBV (2)	174 adults (25–40 y.o., >65 y.o.)	0, 30	0, 7
Weinberger et al., 2018, Front. Immunol. [26]	HBV (3 primary or 1 booster)	41 adults (20–40 y.o., >60 y.o.)	0, 30, 180	0, 1
Rechtien et al., 2017, Cell Rep. [27]	rVSV-ZEBOV (1)	20 adults (18–55 y.o.)	0	0, 1, 3, 7
Vianello et al., 2022, The Lancet Microbe [28]	rVSV-ZEBOV (1)	354 adults (18–55 y.o.)	0	0, 1, 2, 3, 7, 14, 28
Santoro et al., 2021, Vaccines [29]	rVSV-ZEBOV (1)	115 adults (18–65 y.o.)	0	0, 1, 3, 7, 14, 28, 35, 168
Li et al., 2017, Cell [30]	Zostavax (1)	77 adults (25–40 y.o., 60–79 y.o.)	0	0, 1, 3, 7
Popper et al., 2018, J. Infect. Dis. [31]	Dengvaxia (1)	10 adults (<65 y.o.)	0	0, 2, 5, 6, 8, 9, 12, 14, 20, 29, 42, 180
Kim et al., 2022, Cell Reports [32]	Qdenga (2)	20 adults	0, 90	0, 2, 4, 7, 92
Arunachalam et al., 2021, Nature [33]	BNT162b2 (2)	56 adults (19–79 y.o.)	0, 21	0, 1, 7, 21, 22, 23, 28
Papadatou et al., 2023, Vaccines [34]	BNT162b2 (3)	18 adults (28–65 y.o.)	0, 21, 180	21, 24
Lee et al., 2022, Cell Rep. [35]	BNT162b2 (2)	30 adults (I: median 58 y.o., II: >80 y.o.)	I: 0, 35, II: 0, 150	0, 1, 7
Ryan et al., 2023, Cell Reports Med. [36]	BNT162b2 or ChAd/mRNA-1273 or BNT162b2 (3)	102 adults (39 ± 11 y.o.)	0, 28, 164	0, 6, 29, 165
Lee et al., 2022, iScience [37]	ChAd/BNT162b2 (2)	46 adults (median 35 y.o.)	0, 70	0, 3, 7, 70, 73, 77
Zhang et al., 2022, Front. Cell. Infect. Microbiol. [38]	Sinovac CoronaVac (3)	20 adults (24–61 y.o.)	0, 28, 194	0, 14, 42, 208
Bartholomeus et al., 2018, Vaccine [39]	MMR (1)	40 adults (20–30 y.o.)	0	0, 3, 7
Ong et al., 2019, Antiviral Res. [40]	MMR (1)	98 adults (<65 y.o.)	0	0, 1, 3
Noho-Konteh et al. Clin. Infect. Dis. [41]	MV (1)	183 infants (9 m.o.)	0	0, 28
Gomez-Carballa et al., 2021, Front. Immunol. [42]	RV5 (3)	14 infants, children (2–34 m.o.)	0, 30, 60	0, 90
**Studies on vaccines against bacterial and parasitic infections**				
Obermoser et al., 2013, Immunity [43]	PPV23 or TIV (1)	12 adults (18–64 y.o.)	0	0, 1, 3, 7, 10, 14, 21, 28
Li et al., 2014, Nat. Immuol [44]	MPSV4, MCV4 (1)	30 adults (18–45 y.o.)	0	0, 3, 7
O’Connor et al., 2017, Genome Med. [45]	MPSV4 or MCV4/MCV4 (2)	20 adults (30–70 y.o.)	0, 28	0, 7, 28, 35, 36
O’Connor et al., 2020, Mol. Syst. Biol. [46]	4CMenB (1)	181 infants (4 m.o.)	0	0 (0 h, 4 h), 1 (24 h), 3, 7
da Silva Antunes et al. 2021, JCI Insight [47]	Tdap (1)	18 adults	0	0, 1, 3, 7, 14
Kazmin et al., 2017, Proc. Natl. Acad. Sci. [48]	RTS,S (3)	46 adults (mean 30 y.o.)	0, 28, 56	0, 1, 2, 6, 14, 28, 29, 34, 56, 57, 62
Moncunill et al., 2022, Elife [49]	RTS,S (3)	350 infants, children (6–12 w.o., 5–17 m.o.)	0, 30, 60	0, 90
Vahey et al., 2010, J. Infect. Dis. [50]	RTS,S (3)	39 adults (18–45 y.o.)	0, 30, 60	60, 61, 63, 74
van den Berg et al., 2017, Front. Immunol. [51]	RTS,S (3)	117 adults (18–45 y.o.)	0, 30, 60	0, 1, 30, 31, 60, 61, 63, 74

YF-17D: yellow fever vaccine; TIV: inactivated trivalent influenza vaccine; LAIV: live attenuated influenza vaccine; QIV: inactivated quadrivalent influenza vaccine; HBV: vaccine against the hepatitis B virus; rVSV-ZEBOV: vaccine against Ebola virus; Zostavax: vaccine against varicella zoster virus; Dengvaxia, Qdenga: vaccines against Dengue virus; BNT162b2, mRNA-1273, ChAd, Sinovac CoronaVac: vaccines against SARS-CoV-2; MMR: measles–mumps–rubella vaccine; MV: measles vaccine; RV5: live attenuated pentavalent vaccine against rotavirus; PPV23: 23-valent pneumococcal polysaccharide vaccine; MPSV4: meningococcal quadrivalent plain-polysaccharide vaccine; MCV4: meningococcal quadrivalent conjugate vaccine; Tdap: tetanus–diphtheria–pertussis vaccine; RTS,S: vaccine against malaria; 4CMenB: vaccine against meningococcus B. y.o.: years old; m.o.: months old, w.o.: weeks old.

## Data Availability

As this is a review article, no data were collected from patients.
